# Effects of different grassland use patterns on soil bacterial communities in the karst desertification areas

**DOI:** 10.3389/fmicb.2023.1208971

**Published:** 2023-08-31

**Authors:** Yongkuan Chi, Shuzhen Song, Kangning Xiong

**Affiliations:** ^1^School of Karst Science, Guizhou Normal University, Guiyang, China; ^2^Guizhou Engineering Laboratory for Karst Desertification Control and Eco-Industry, Guiyang, China

**Keywords:** artificial grassland, soil microorganism, sustainable utilization, karst desertification, Illumina sequencing

## Abstract

Soil bacteria are closely related to soil environmental factors, and their community structure is an important indicator of ecosystem health and sustainability. A large number of artificial grasslands have been established to control rocky desertification in the karst areas of southern China, but the influence of different use patterns on the soil bacterial community in artificial grasslands is not clear. In this study, three grassland use patterns [i.e., grazing (GG), mowing (MG), and enclosure (EG)] were used to investigate the effects of different use patterns on the soil bacterial community in artificial grassland by using 16S rDNA Illumina sequencing and 12 soil environmental indicators. It was found that, compared with EG, GG significantly changed soil pH, increased alkaline hydrolyzable nitrogen (AN) content (*P* < 0.05), and decreased soil total phosphorus (TP) content (*P* < 0.05). However, MG significantly decreased the contents of soil organic carbon (SOC), total phosphorus (TP), available nitrogen (AN), ammonium nitrogen (NH_4_^+^-N), β-1,4-glucosidase (BG), and N-acetyl-β-D-glucamosonidase (NAG) (*P* < 0.05). The relative abundance of chemoheterotrophy was significantly decreased by GG and MG (*P* < 0.05). GG significantly increased the relative abundance of Acidobacteria and Gemmatimonadota (*P* < 0.05) and significantly decreased the relative abundance of Proteobacteria (*P* < 0.05), but the richness index (Chao 1) and diversity index (Shannon) of the bacterial community in GG, MG, and EG were not significantly different (*P* > 0.05). The pH (R^2^ = 0.79, *P* = 0.029) was the main factor affecting the bacterial community structure. This finding can provide a scientific reference for ecological restoration and sustainable utilization of grasslands in the karst desertification areas.

## Introduction

Soil microorganisms, as the engine of the biogeochemical cycle, play a central role in regulating soil fertility, plant growth, and climate change (Nelson et al., 2016; He et al., 2022; Ma L. N. et al., 2022). They maintain the sustainable development of the soil ecosystem. Changes in the composition and abundance of soil microbial communities are important biological indicators for measuring soil fertility as they directly influence the composition and transformation of soil nutrients and indicate the level of ecosystem health (Baranova et al., 2019). Regulating the diversity and function of soil microbial can enhance terrestrial ecosystem services and promote the restoration of degraded ecosystems (Yu et al., 2022). Soil microbes play the role of “changer maker” in restoring degraded soil functions (Coban et al., 2022), so studying the links between microbial community functions and ecosystem changes has been a challenge for soil microbiology research (Fierer, 2017; Ma Q. X. et al., 2022). Because changes in microbial communities have an impact on community function and their sensitivity to adversity, it is unclear how microbial community structure responds to anthropogenic environmental perturbations, similar to different utilization patterns.

Soil bacteria are the most abundant and widely distributed among soil microorganisms, accounting for 70%−90% of all soil microorganisms, and their various metabolic activities are closely related to soil environmental factors and are extremely sensitive to environmental changes (Liu et al., 2015; Xue et al., 2017; Sui et al., 2019; Lu et al., 2022; Qiu et al., 2023). Soil bacterial communities are not only influenced by the climatic environment but also directly and indirectly affected by changes in vegetation (de Vries et al., 2017; Mod et al., 2021). There is evidence that bacterial communities respond directly or indirectly to anthropogenic changes in the soil environment (Hermans et al., 2020; Li et al., 2023). It was found that the composition of soil bacterial communities is closely related to the type of land use and that the type of land use can be correctly determined with an accuracy of up to 85% (Hermans et al., 2020). Some studies also found that heavily managed soils contain different bacterial communities compared to unmanaged soils (Drenovsky et al., 2010), and the type of land use correlates with changes in the composition of bacterial communities (Plassart et al., 2019). More specifically, management practices such as fertilization, establishment of artificial plants, or grazing have been shown to affect soil microbial communities (Fierer et al., 2012; Figuerola et al., 2015; Cassman et al., 2016). Overall, the composition of bacterial communities is strongly influenced by changes in the soil environment, many of which are a direct result of land-use change (Hermans et al., 2020). Therefore, by investigating the composition and variation of soil bacterial communities, we can better predict and control degraded ecosystem changes to realize the improvement and sustainable development of degraded ecosystems (Sun et al., 2022).

Karst landscapes account for ~15% of the world's total land area (Xiong et al., 2017; Garousi et al., 2021). Among them, South China Karst is one of the three major concentrated and continuous karst distribution areas in the world (Xiong et al., 2016; Zhang J. X. et al., 2021). Due to global climate change and unreasonable land use, the karst ecosystem in southern China has been severely degraded, and the problem of rocky desertification is prominent (Jiang et al., 2014), seriously threatening the ecological security and socioeconomic sustainable development of the region (Li S. L. et al., 2020). Desertification has long been a major economic, social, and environmental issue of concern to many countries and regions of the world (Oldroyd and Leyser, 2020). Karst desertification, which is similar to desertified landscapes, has attracted much attention from the international community (Xiong et al., 2002). It has been shown that the establishment of artificial grasslands is an important initiative to rapidly repair the damaged ecological environment of rocky desertification (Wang et al., 2019), which is of great significance to promote ecological reconstruction and economic development (Fang et al., 2018; Xin et al., 2020; Bai and Cotrufo, 2022). However, due to the different utilization of artificial grassland, desertification may change the microbial community structure formed by long-term evolution and then change the soil microbial–soil–plant nutrient relationship (Pei et al., 2021), ultimately having a profound effect on grassland ecosystems and their functions (Liu et al., 2022).

Since 1999, a large-scale special project of grain for green and vegetation restoration to control rocky desertification has been implemented in the karst areas of southern China. According to statistics, the area of ecological measures dominated by establishing artificial grassland has exceeded 10,000 km^2^ (Liu et al., 2021), and good social, economic, and ecological benefits have been achieved. Although many previous studies have been carried out on the response of grassland soil bacteria to land-use change in the karst desertification area, such as soil microbial communities (Li et al., 2018; Huang et al., 2020; Song et al., 2022; Barber et al., 2023), limitation of soil microbial resources (Chen et al., 2019; Sun et al., 2021; Qian et al., 2022; Wu et al., 2022; Soozandehfar et al., 2023), and community structures and functions (Wang D. et al., 2022). However, systematic studies on the response of soil bacterial communities in artificial grasslands to different use patterns are obviously lacking, especially for artificial grasslands in the karst desertification areas, which are at the stage of sporadic exploration. Therefore, there is an urgent need to clarify the response mechanisms of soil bacterial communities to different grassland utilization modes to help us comprehensively understand and predict the effects of external disturbances on grassland ecosystems and their future trends (Feng et al., 2022). In this study, it was hypothesized that different utilization patterns of artificial grassland in the karst desertification control area will change the structure, richness, and diversity of the soil bacterial community. To address the above hypotheses, this study analyzed the effects of different grassland use patterns on soil bacterial communities by using 16S rDNA Illumina sequencing and soil chemical properties analysis technology. The objectives were to (1) investigate changes in soil chemical index and soil enzyme activity under different grassland utilization patterns; (2) evaluate the influence of different use patterns on soil bacteria structure and diversity in artificial grassland; and (3) determine the relationship between soil bacterial community and environmental factors so as to provide theoretical support for ecological restoration and sustainable utilization of grassland in the karst desertification control areas in southern China.

## Materials and methods

### Study area

The study area is located in Salaxi Town, Qixingguan District, Bijie City, Guizhou Province (105°02′01″-105°08′09″E, 27°11′36″-27°16′51″N), which is a typical karst plateau mountain area with light-to-moderate karst desertification. The karst desertification area is 55.931 km^2^, accounting for 64.93% of the total area of the demonstration area. The study area belongs to the subtropical monsoon humid climate, with an average altitude of 1,800 m, an annual average temperature of about 12°C, a frost-free period of 245 days, an average annual sunshine duration of 1,360 h, an annual average rainfall of 984.4 mm, and precipitation concentrated from June to September. The soil is zonal calcareous soil. The vegetation is dominated by *Cyclobalanopsis glauca, Pyracantha fortuneana, Rhododendron simsii, Juglans regia, Rosa roxburghii, Artemisia lavandulaefolia, Chenopodium glaucum, Clinopodium chinense, Plantago asiatica, Stellaria media, Digitaria sanguinalis*, and *Polygonum hydropiper*.

### Experiment design and sampling

During the implementation of China's “13th Five-Year” National Key Research & Development Project, our research team established artificial grassland in the study area in mid-April 2012 to restore the damaged karst desertification ecosystem. The artificial grassland was sown with *Lolium perenne*+*Trifolium repens*+*Dactylis glomerata*, and it was planted according to the seed ratio of 2:2:1. After the establishment of artificial grassland, free grazing was the main practice, and the carrying capacity was sheep unit/600m^2^. To reveal the effect of artificial grassland on soil microbial bacterial communities under different utilization patterns, we set up grazing treatment (GG), mowing treatment (MG), and enclosure treatment (EG) for a comparative study in August 2019. The area of each treatment plot was ~3,000 m^2^, and three replicates were set. The average number of grazing animals on each plot was 5 (basically consistent with the local grazing situation). The grazing livestock were approximately 1-year-old Guizhou semi-fine wool sheep. Except for extreme weather, the grazing period was ~300 days per year. The stubble height in the mowing grassland was ~5 cm, and the mowing was carried out according to the normal forage phenology period or when the mowing height was reached. The enclosure grassland was not used in any way.

In mid-August 2021, three 10 m × 10 m sampling plots were set in each test site (nine sampling plots in total), and the distance between plots and their boundaries was >10 m. In each sampling plot, 15 sampling points (~3 cm away from the base of the plant) were evenly spaced using an “S”-shaped multi-point sampling method. After removing the litter layer from the surface, soil samples were collected from the surface layer (0–10 cm) using a soil auger. To reduce spatial heterogeneity, soil samples from 15 sampling points were mixed into 1 sample, and a total of 9 soil samples were obtained. The soil samples were removed from impurities and then divided into three parts. One part was placed in 15-ml sterilized centrifuge tubes and stored in liquid nitrogen for transport back to the laboratory, and these samples were stored in a refrigerator at −80°C for 16s rDNA analysis (Li H. N. et al., 2020; Sardar et al., 2021a,b). One part was placed in a sealed plastic bag and taken back to the laboratory for the determination of the soil enzyme activity. Another part of the soil samples was air-dried in the room and then passed through a 2-mm sieve to determine soil properties.

### Determination of soil properties

In this study, 12 indices of soil samples were determined, namely pH, soil organic carbon (SOC), total nitrogen (TN), total phosphorus (TP), available nitrogen (AN), available phosphorus (AP), nitrate nitrogen (NO_3_^−^-N), ammonium nitrogen (NH_4_^+^-N), β-1,4-glucosidase (BG), N-acetyl-β -D-glucosaminidase (NAG), acid phosphatase (LCP), and leucine aminopeptidase (LAP). Soil pH was determined in suspension with a water-to-soil ratio of 2.5:1 using a pH meter (PHC-3C, Leici, Shanghai, China). SOC, TN, TP, AN, and AP were determined using the method described by Bao (2000). SOC and TN were determined using an automatic elemental analyzer (FlashSmart, Thermo Fisher, USA). TP, AN, and AP were determined on a continuous flow analyzer (Flowsys, Systea, Italy). Nitrogen and ammonium nitrogen in the soil were extracted with potassium chloride solution according to ISO standards and determined with a continuous flow analyzer (Flowsys, Systea, Italy). Soil enzyme activity was analyzed for BG, NAG, LCP, and LAP according to the method of Jiao et al. (2022) and determined with UV-visible spectrophotometer (Specord 200 Plus, Analytik, Germany), with active units expressed as IUg^−1^ units.

### DNA collection and high-throughput sequencing

The DNA was extracted with the TGuide S96 Magnetic Soil DNA Kit (Tiangen Biotech Beijing Co., Ltd.) according to manufacturer instructions. The DNA concentration of the samples was measured with the Qubit dsDNA HS Assay Kit and Qubit 4.0 Fluorometer (Invitrogen, Thermo Fisher Scientific, Oregon, USA). The 338F: 5′- ACTCCTACGGGAGGCAGCA-3′ and 806R: 5′- GGACTACHVGGGTWTCTAAT-3′ universal primer set was used to amplify the V3-V4 region of 16S rRNA gene from the genomic DNA extracted from each sample. Both the forward and reverse 16S primers were tailed with sample-specific Illumina index sequences to allow for deep sequencing. The PCR was performed in a total reaction volume of 10 μl: DNA template 5-50 ng, ^*^Vn F (10μM) 0.3 μl, ^*^Vn R (10μM) 0.3 μl, KOD FX Neo Buffer 5 μl, dNTP (2 mM each) 2 μl, KOD FX Neo 0.2 μl, and ddH2O up to 10 μl. Vn F and Vn R are selected according to the amplification area: initial denaturation at 95°C for 5 min, followed by 25 cycles of denaturation at 95°C for 30 s, annealing at 50°C for 30 s, and extension at 72°C for 40 s, and a final step at 72°C for 7 min. A total of PCR amplicons were purified with Agencourt AMPure XP beads (Beckman Coulter, Indianapolis, IN) and quantified using the Qubit dsDNA HS Assay Kit and Qubit 4.0 Fluorometer (Invitrogen, Thermo Fisher Scientific, Oregon, USA). After the individual quantification step, amplicons were pooled in equal amounts. For the constructed library, use Illumina NovaSeq 6000 (Illumina, Santiago CA, USA) for sequencing.

### Statistical analysis

According to the quality of single nucleotide, raw data were primarily filtered by Trimmomatic (Version 0.33) (Edgar, 2013). The identification and removal of primer sequences were processed by Cutadapt (Version 1.9.1) (Callahan et al., 2016). PE reads obtained from previous steps were assembled by USEARCH (Version 10) (Segata et al., 2011) and followed by chimera removal using UCHIME (Version 8.1) (Quast et al., 2013). The high-quality reads generated from the above steps were used in the following analysis. Sequences with similarity ≥ 97% were clustered into the same operational taxonomic unit (OTU) by USEARCH (Version 10.0) (Edgar, 2013), and the OTUs with a relative abundance of < 0.005% were filtered. Taxonomy annotation of the OTUs was performed based on the Naive Bayes classifier in QIIME2 (Bolyen et al., 2019) using the SILVA database (release 132) (Quast et al., 2013) with a confidence threshold of 70%. The alpha diversity was calculated and displayed by the QIIME2 and R software, respectively. Beta diversity was determined to evaluate the degree of similarity of microbial communities from different samples using QIIME.

### Data analysis

The one-way analysis of variance (ANOVA) and multiple comparisons (Tukey's pairwise comparisons) were used to analyze the effects of different use patterns on soil properties, relative abundance of bacterial phylum, and bacterial community richness and diversity indices (SPSS 19.0, Chicago, IL, USA). The data were log-transformed to fit the normal distribution of the data. The Chao 1 and Shannon indices of alpha diversity were calculated using the Mothur software based on OTU information, which was used to reflect the richness and diversity of soil microbial communities. The common and unique OTUs and soil bacterial structure at the phylum level among grassland samples under different utilization patterns were presented using the Vegan software package in R (Chen and Boutros, 2011). PCoA analysis and similarity test (ANOSIM) based on Bray–Curtis distance measure were used to compare the beta diversity of soil bacteria in grassland under different use patterns.

The variance inflation factor (VIF) was used to filter the multicollinearity of environmental factors when VIF >10. Redundancy analysis (RDA) was used to analyze the correlation between the selected environmental factors and soil microbial communities. To test the correlation of environmental factors with bacterial community composition, we performed a Monte Carlo permutation test of soils using the R “ecodist” package, and the Bray–Curtis index served as the dissimilarity metric. The relationships between environmental factors and soil bacterial structure at the phylum level were analyzed using the Spearman correlation heatmap of the Vegan software package in R. The Functional Annotation of Prokaryotic Taxa database 12 (FAPROTAX, v1.2.3) was used to predict the ecological function of soil bacteria (Sansupa et al., 2021).

## Results

### Soil chemical properties and soil enzyme activities of grassland under different use patterns

**Figure 1:**
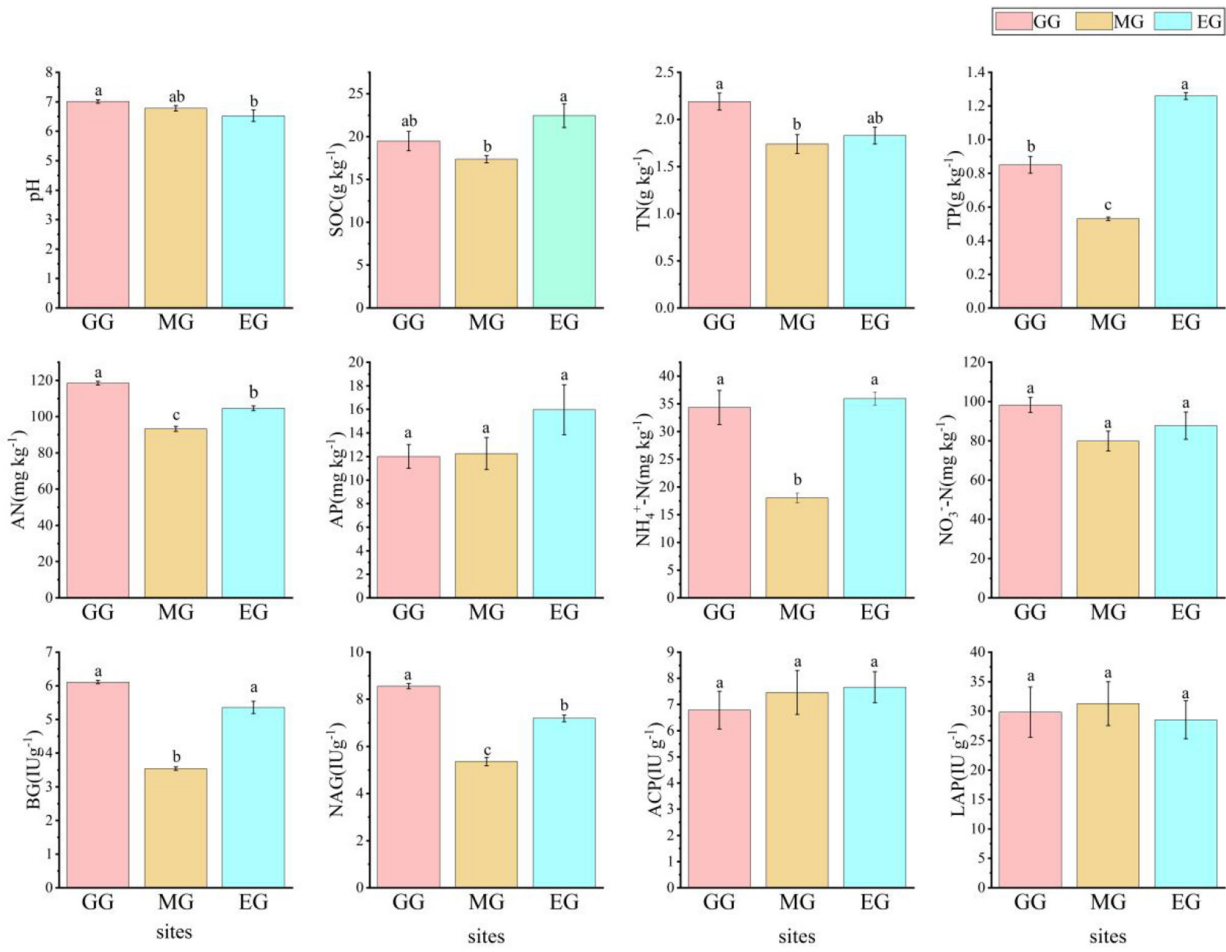
Soil chemical properties and soil enzyme activity under different grassland use patterns [different lowercase letters indicate the significant differences between different treatments (*P* < 0.05, *n* = 3)]. SOC, soil organic carbon; TN, total nitrogen; TP, total phosphorus; AN, available nitrogen; AP, available phosphorus; NO_3_^−^-N, nitrate nitrogen; NH_4_^+^-N, ammonium nitrogen; BG, β-1,4-glucosidase; NAG, N-acetyl-β -D-glucosaminidase; LCP, acid phosphatase; LAP, leucine aminopeptidase; GG, grazing grassland; MG, mowing grassland; EG, enclosure grassland.

Different use patterns have different effects on soil chemical properties and soil enzyme activity in grassland (Figure 1). Compared to EG, GG significantly changed soil pH, increased the soil AN content (*P* < 0.05), and decreased the soil TP content and NAG content (*P* < 0.05). MG significantly decreased the contents of SOC, TP, AN, NH_4_^+^-N, BG, and NAG (*P* < 0.05). However, GG and MG had no significant effect on pH, SOC, AP, NO_3_^−^-N, ACP, and LAP (*P* > 0.05).

### Alpha diversity of grassland under different use patterns

**Figure 2:**
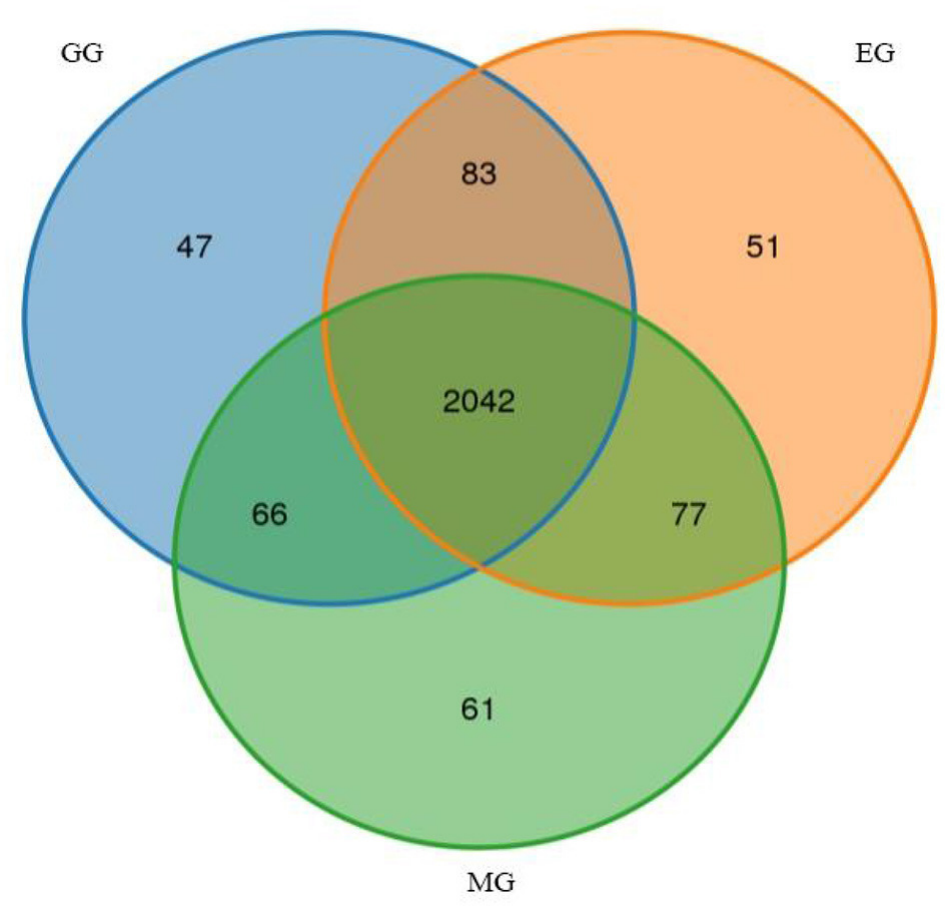
Venn diagram of unique and shared bacterial OTUs under different grassland use patterns (at the 3% evolutionary distance). GG, grazing grassland; MG, mowing grassland; EG, enclosure grassland.

A total of 720,323 raw reads were detected in all samples, generating an average of 716,180 clean reads and 695,633 effective reads, and the average sequence length was 420.44 bp (Supplementary Table S1). After species annotation, the average sequence obtained from all samples was 56,723, ranging from 49,679 to 60,248 (Supplementary Table S2). A total of 2,427 OTUs were obtained, which were divided into 26 phyla, 54 classes, 153 orders, 288 families, 505 genera, and 575 species (Supplementary Table S3). We illustrated the similarities and differences between OTUs from different treatments using the Venn diagram (Figure 2). The numbers of unique OTUs in the GG, MG, and EG were 47, 61, and 51, respectively, and the number of shared OTUs was 2,042. The Chao 1 and Shannon indices represent the richness and diversity of bacterial communities, which are used to study the alpha diversity of soil microbial bacteria in grasslands under different utilization patterns (Table 1). There was no significant difference in bacterial community richness (Chao 1) and diversity (Shannon) among treatments. This indicated that grazing and mowing did not significantly change the richness and diversity of the bacterial communities compared to EG.

**Table 1:** Effect of different utilization methods on bacterial alpha diversity in grassland soil.

**Treatment**	**OUT number**	**Chao 1 index**	**Shannon index**	**Coverage (%)**
GG	1991.67 ± 28.36a	2133.37 ± 69.74a	9.54 ± 0.06a	99.65 ± 0.05a
MG	1913.00 ± 118.49a	2106.36 ± 120.53a	9.31 ± 0.29a	99.54 ± 0.06a
EG	1973.33 ± 56.72a	2129.18 ± 49.44a	9.27 ± 0.32a	99.60 ± 0.05a

Mean value (mean ± standard deviation, *n* = 3), different lowercase letters indicate the difference between treatments reaching a significant level (*P* < 0.05). GG, grazing grassland; MG, mowing grassland; EG, enclosure grassland.

### Bacterial community structure of grassland under different use patterns

**Figure 3:**
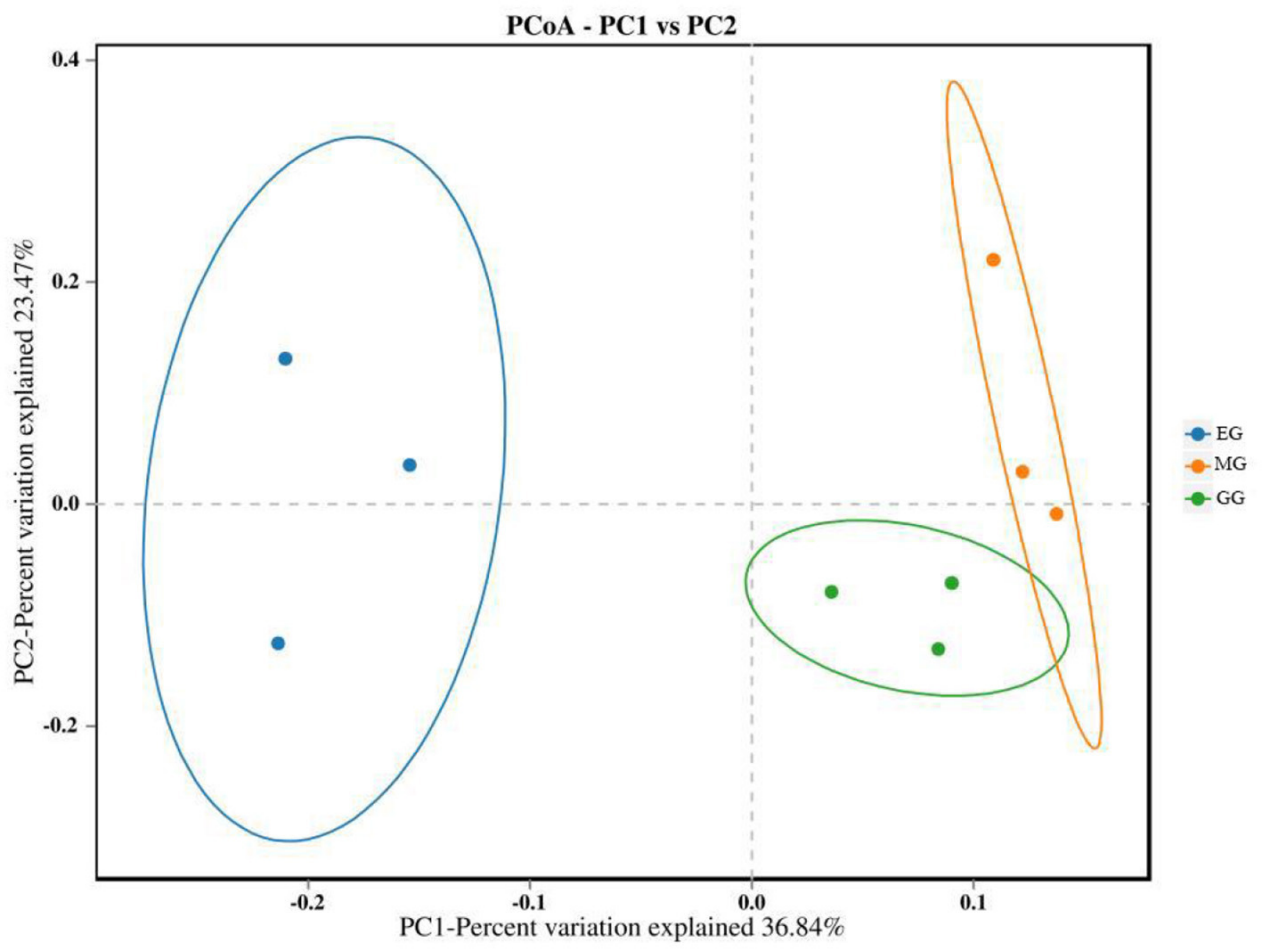
PCoA of bacterial community composition under different grassland use patterns. GG, grazing grassland; MG, mowing grassland; EG, enclosure grassland.

**Figure 4:**
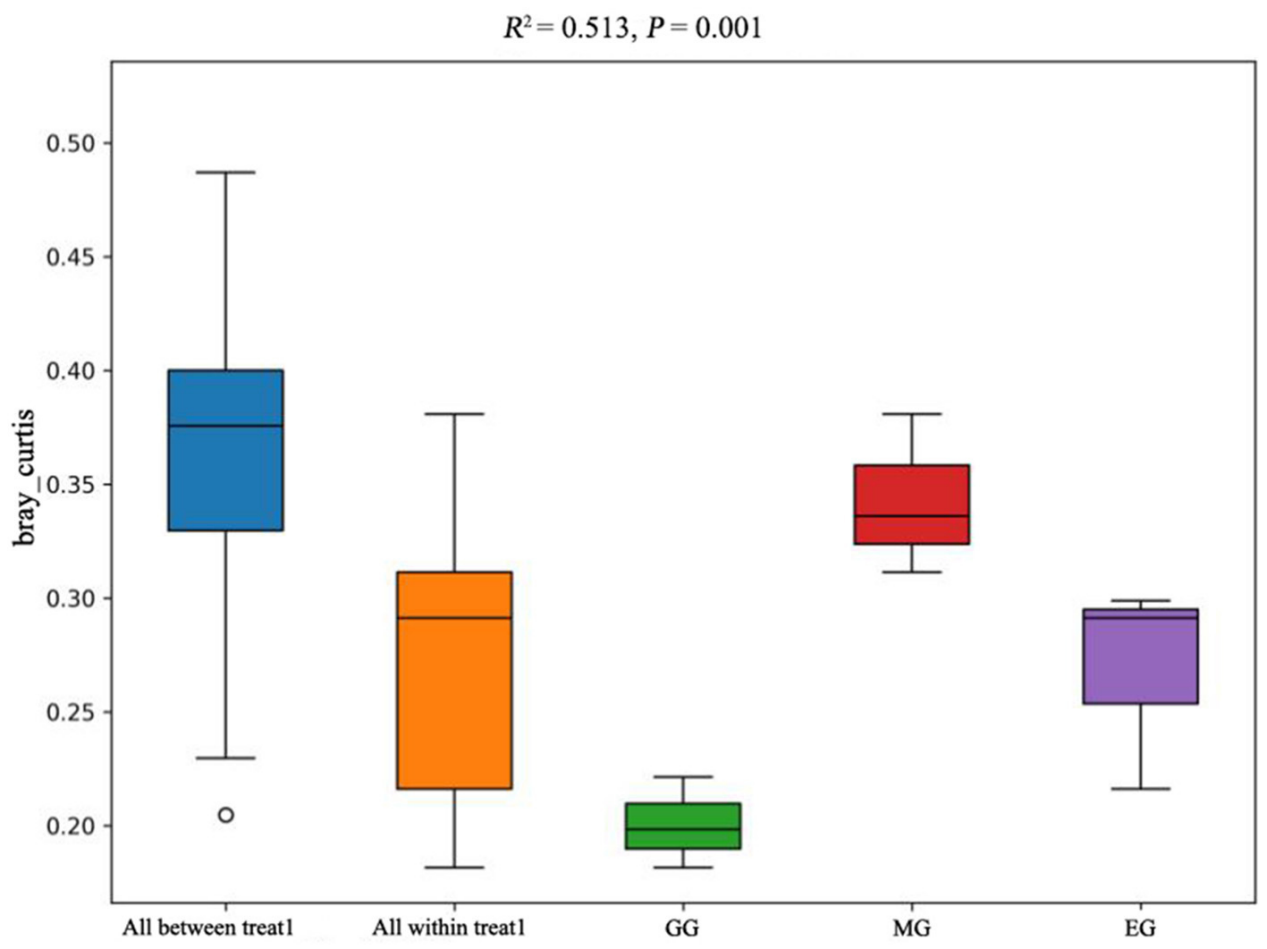
ANOSIM of soil bacterial community composition under different grassland use patterns based on the Bray–Curtis distance metric. GG, grazing grassland; MG, mowing grassland; EG, enclosure grassland.

The PCoA of soil bacterial communities under different use patterns was analyzed using the Bray–Curtis distance metric. The PCoA results showed that the bacterial communities in different treatments were largely separated (Figure 3), and the ANOSIM test results based on the Bray–Curtis distance metric also confirmed this finding (*R*^2^ = 0.513, *P* = 0.001) (Figure 4), which indicated that different utilization patterns significantly changed the soil bacterial community structure in grassland.

**Figure 5:**
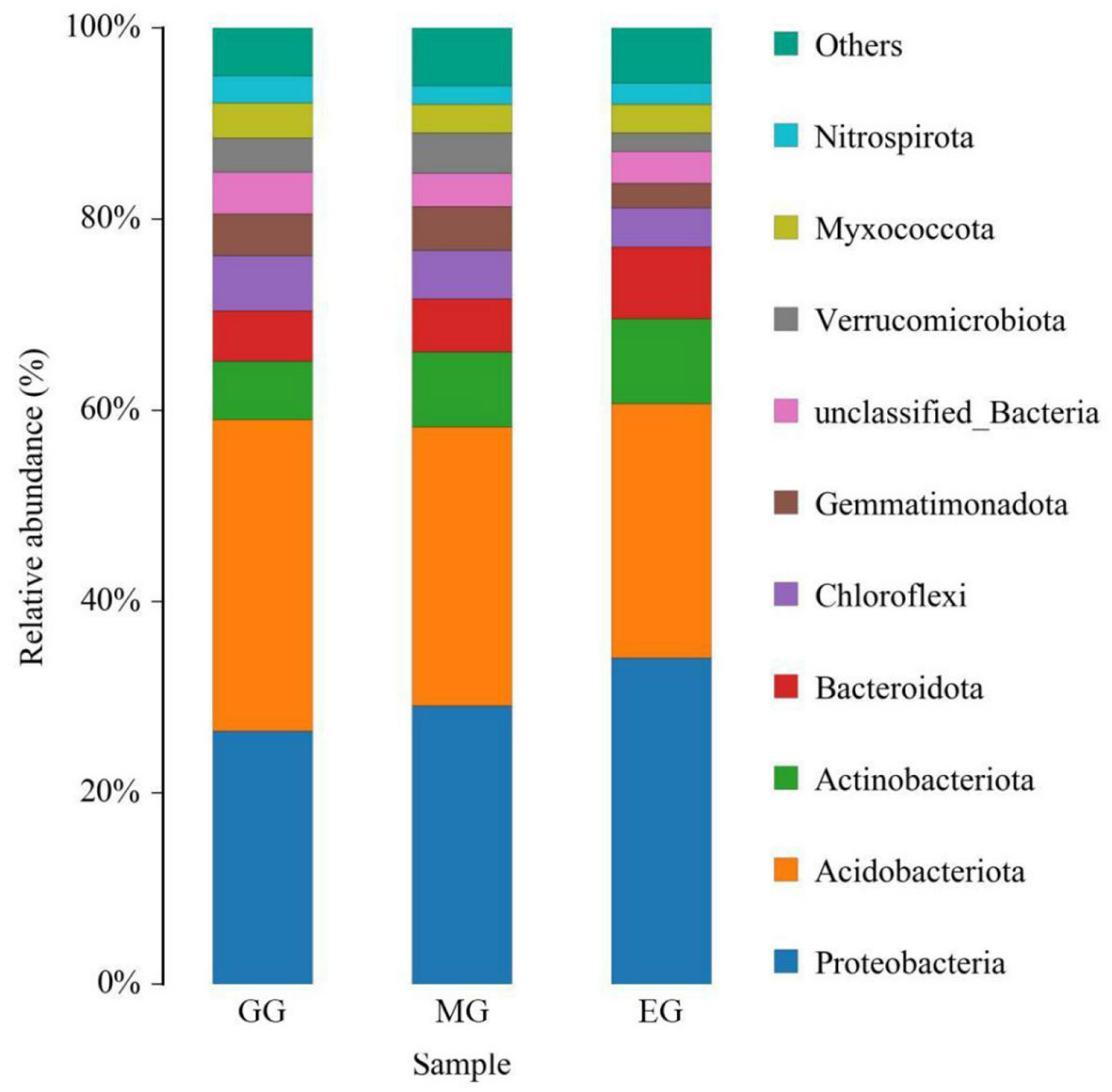
Relative abundance of soil bacterial phylum under different grassland use patterns (relative abundance>1%). GG, grazing grassland; MG, mowing grassland; EG, enclosure grassland.

At the phylum level, a total of 10 soil bacterial phyla with a relative abundance of >1% were obtained (Figure 5). The order was as follows: Proteobacteria (29.88%), Acidobacteriota (29.46%), Actinobacteriota (7.61%), Bacteroidota (6.13%), Chloroflexi (4.97%), Gemmatimonadota (3.83%), unclassified_Bacteria (3.71%), Verrucomicrobiota (3.25%), Myxococcota (3.25%), and Nitrospirota, (2.33%). Among them, Proteobacteria and Acidobacteriota were the dominant bacteria. The results of ANOVA showed that different use patterns had a significant effect on the relative abundance of Proteobacteria, Acidobacteriota, Gemmatimonadota, and Nitrospirota (*P* < 0.05) but no significant effect on other phyla (*P* > 0.05). Compared with EG, the GG significantly increased the relative abundance of Acidobacteriota and Gemmatimonadota (*P* < 0.05) but significantly decreased the relative abundance of Proteobacteria (*P* < 0.05) and had no significant difference with MG (*P* > 0.05). Compared to GG, MG significantly increased the relative abundance of Nitrospirota (*P* < 0.05) but had no significant difference with EG (*P* > 0.05). However, there were no significant differences between the three treatments at the family and genus levels (Supplementary Table S4).

### Relationship between soil bacterial communities and environmental factors

**Figure 6:**
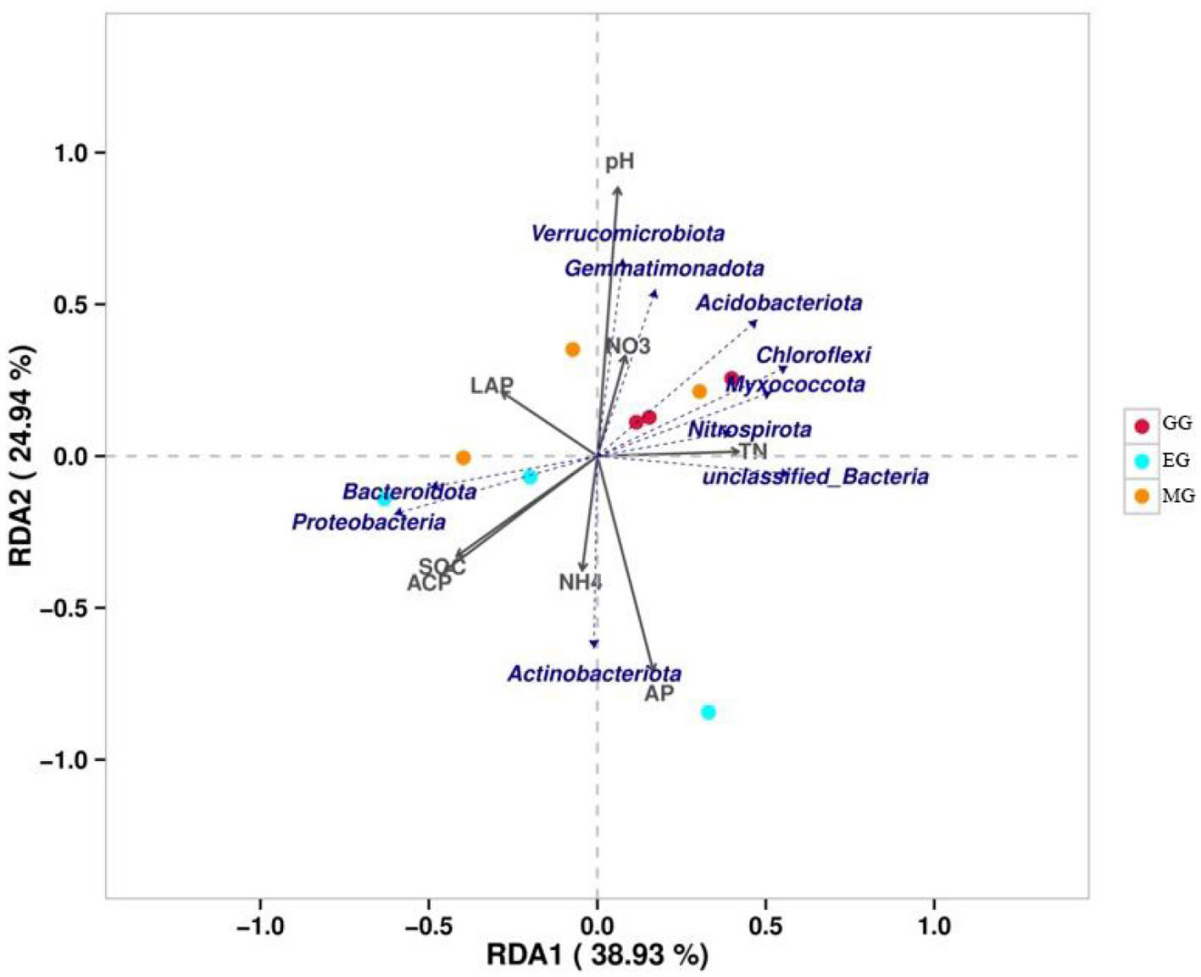
Redundancy analysis of soil properties (gray arrows) and soil microbial structure (blue arrows) at the phylum level. SOC, soil organic carbon; TN, total nitrogen; AP, available phosphorus; NO_3_, nitrate nitrogen; NH_4_, ammonium nitrogen; LCP, acid phosphatase; LAP, leucine aminopeptidase; GG, grazing grassland; MG, mowing grassland; EG, enclosure grassland.

**Figure 7:**
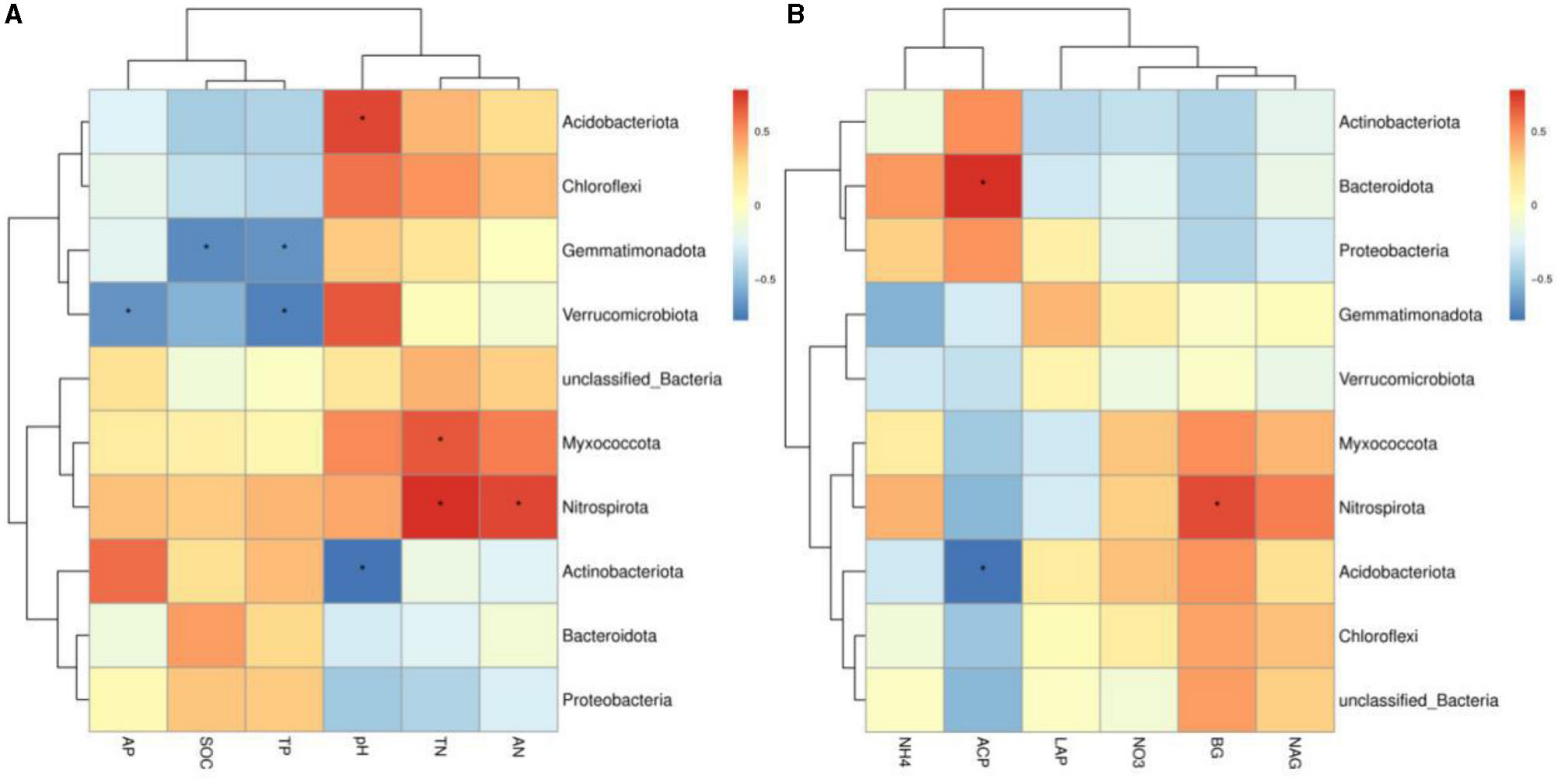
**(A, B)** Heatmap of the correlation between environmental factors and soil bacterial community at the phylum level (relative abundance >1%). Spearman's correlation coefficients are shown in different colors, and the legend on the right shows the color range of the different correlation coefficients (*P*-values: ^*^ < 0.05). SOC, soil organic carbon; TN, total nitrogen; TP, total phosphorus; AN, available nitrogen; AP, available phosphorus; NO_3_^−^-N, nitrate nitrogen; NH_4_^+^-N, ammonium nitrogen; BG, β-1,4-glucosidase; NAG, N-acetyl-β -D-glucosaminidase; LCP, acid phosphatase; LAP, leucine aminopeptidase; GG, grazing grassland; MG, mowing grassland; EG, enclosure grassland.

The variance inflation factor (VIF) was used to filter the multicollinearity of environmental factors (Supplementary Table S5). Redundancy analysis of soil microbial structure at the phylum level and soil properties indicated that the first and second axes explain 38.93% and 24.94% of the variation in the bacterial community, respectively (Figure 6). Further analysis by Monte Carlo test showed that pH (*R*^2^ = 0.79, *p* = 0.029) had a higher correlation with the bacterial community composition than other environmental factors, which was the main factor affecting the bacterial community structure (Table 2). The related heatmap was used to reveal the relationship between environmental factors and soil microbial structure at the phylum level (relative abundance> 1%) (Figures 7A, B). The results showed that there was a significantly positive correlation between pH and Acidobacteriota (*P* < 0.05) but a significantly negative correlation with Actinobacteria (*P* < 0.05). SOC and TP have a significantly negative correlation with Gemmatimonadota (*P* < 0.05). AP and TP have a significantly negative correlation with Verrucomicrobiota (*P* < 0.05). TN, AN, and BG have a significantly positive correlation with Nitrospirota (*P* < 0.05). TN has a significantly positive correlation with Myxococcota (*P* < 0.05). ACP has a significantly positive correlation with Bacteroidota (*P* < 0.05) but a significantly negative correlation with Acidobacteriota (*P* < 0.05).

**Table 2:** Results of analysis by the Monte Carlo test.

**Environmental factors**	***R* ^2^**	***P*-value**
pH	0.79	0.029
SOC	0.28	0.389
TN	0.18	0.597
AP	0.54	0.082
NH_4_^+^-N	0.14	0.657
NO_3_^−^-N	0.12	0.736
ACP	0.35	0.249
LAP	0.13	0.717

SOC, soil organic carbon; TN, total nitrogen; AN, available nitrogen; AP, available phosphorus; NO_3_^−^-N, nitrate nitrogen; NH_4_^+^-N, ammonium nitrogen; LCP, acid phosphatase; LAP, leucine aminopeptidase.

**Figure 8:**
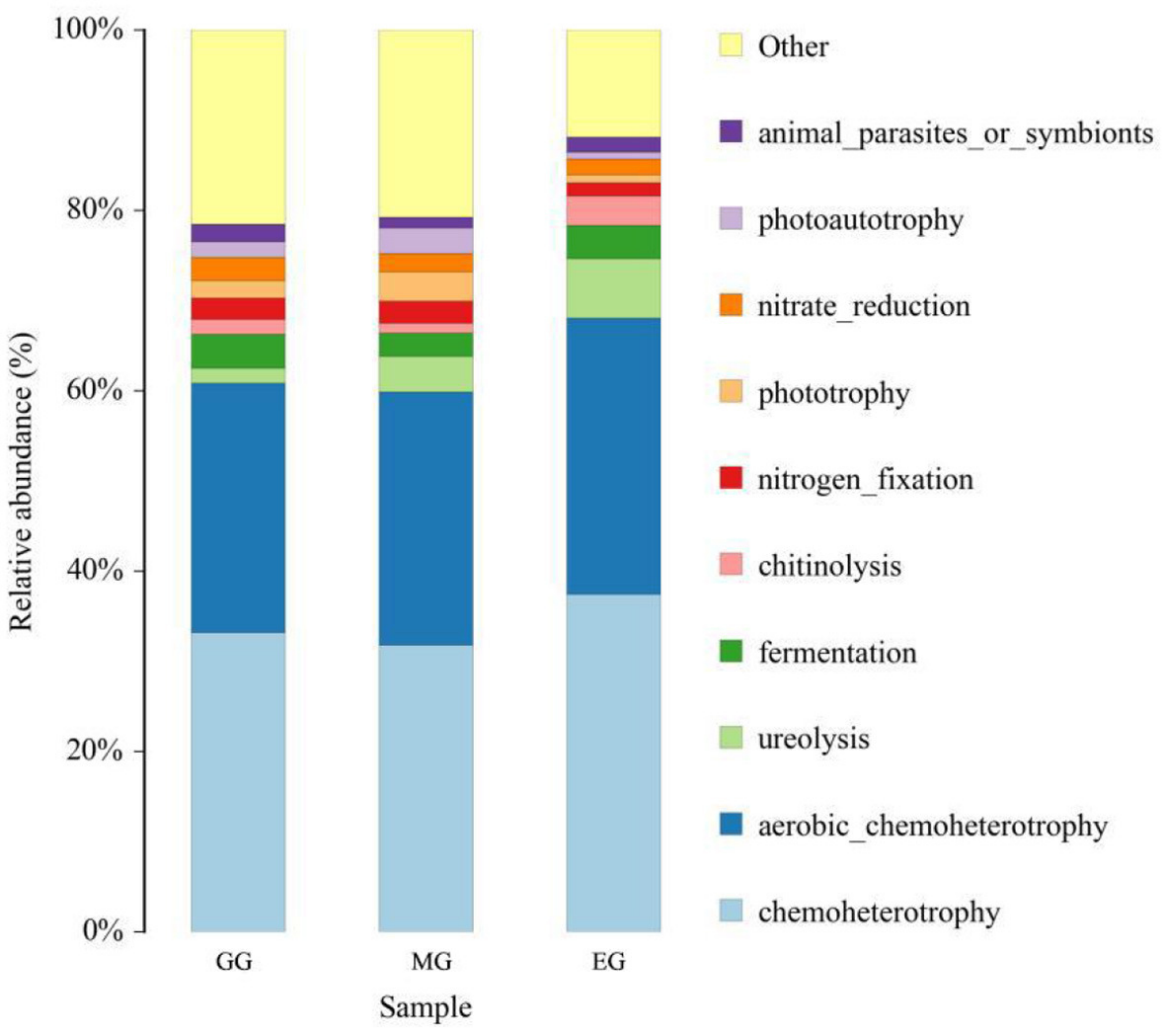
Relative abundance of soil bacterial community for ecological function under different grassland use patterns. GG, grazing grassland; MG, mowing grassland; EG, enclosure grassland.

FAPROTAX software was used to predict the ecological function of soil bacteria in different utilization patterns (Figure 8), and a total of 46 functional groups were obtained. In all the ecological function groups, the relative abundance of chemoheterotrophy, aerobic chemoheterotrophy, ureolysis decomposition, and fermentation bacteria was relatively high, with an average abundance of 34.14%, 28.80%, 4.07%, and 3.34%, respectively. The results of ANOVA showed that GG and MG significantly decreased the relative abundance of chemoheterotrophy compared to EG (*P* < 0.05), but there was no significant difference between GG and MG (*P* > 0.05). There was no significant difference in the relative abundance of aerobic chemoheterotrophy, ureolysis, and fermentation bacteria between GG, MG, and EG (*P* > 0.05).

## Discussion

### Effects of different use patterns on soil chemical properties and soil enzyme activity in the grassland

Artificial grassland is an ecosystem under human disturbance, and its quality and function were good or bad, which often cannot be evaluated from macroscopic perspectives such as vegetation cover (Wang et al., 2020), while soil chemical properties and soil microorganisms can better evaluate the quality of grassland under different use patterns (Baptistella et al., 2020; Song et al., 2022). In this study, there were significant differences in the soil chemistry of artificial grassland under different use patterns after 2 years. It has been shown that grazing can increase the soil pH of artificial grassland (Wei et al., 2022), which is consistent with the results of this study. This may be related to the excreta of grazing livestock. As the excreta mature, the soil animal community increases and the cation cycle in the grassland ecosystem accelerates, thus offsetting soil acidification and resulting in a trend toward weak alkalinity of the soil. As MG and EG were not disturbed by livestock, the difference in soil pH was not significant. In this study, compared with EG, GG significantly increased soil TN and AN content (*P* < 0.05) but significantly decreased TP content (*P* < 0.05), which is consistent with the results of Zheng et al. (2022) and Guan et al. (2022). This may be due to nitrogen enrichment in the grassland ecosystem caused by grazing and increased soil phosphorus supply capacity through activation of mineral-bound phosphorus, but long-term grazing leads to depletion of the soil phosphorus pool (Wang R. Z. et al., 2022). Compared with EG, MG significantly decreased the content of soil SOC, TP, AN, NH_4_^+^-N, NO_3_^−^-N, BG, and NAG (*P* < 0.05), indicating that MG was not conducive to the accumulation of TN in soil, and at the same time, the available carbon and nitrogen sources by microorganisms were reduced, which decreased the activity of microorganisms and subsequently decreased the enzyme activity, which is consistent with the research results of Wang et al. (2023). GG and MG had no significant effect on soil AP, ACP, and LAP (*P* > 0.05). This indicates that the available phosphorus did not obviously respond to GG and MG, but the AP content in EG was relatively higher, which may be related to its higher SOC and more complete decomposition.

### Effects of different use patterns on bacterial community structure and alpha diversity in the grassland

As an important component of the soil system, the structure and diversity of soil microorganisms play an important role in the stability of the grassland ecosystem (Sennett et al., 2023). The dominant taxa in soil bacteria have important functions in grassland ecosystems (Ou et al., 2023). In this study, the results of PCoA showed that there were significant differences among the groups tested by ANOSIM (*R*^2^ = 0.513, *P* = 0.001), and the soil bacterial communities of grasslands under different utilization patterns were basically separated, indicating that the different use patterns significantly changed the bacterial community structure. The results of ANOVA showed that the relative abundance of Proteobacteria, Acidobacteriota, Gemmatimonadota, and Nitrospirota was significantly affected by the different use patterns (*P* < 0.05). Proteobacteria, Acidobacteriota, Gemmatimonadota, and Nitrospirota are considered potential decomposer nutrients in the soil (Berlemont and Martiny, 2013). Proteobacteria can make full use of soil carbon and grow rapidly under the condition of sufficient soil carbon (Zhang Y. T. et al., 2020). They can also use nutrients produced by the decomposition of organic matter for growth and metabolism (Huang et al., 2022). Gemmatimonadota are believed to promote the rapid decomposition of organic matter such as litter, animal, and plant remains in the soil by decomposing organic matter (Lozupone and Knight, 2008). Proteobacteria and Gemmatimonadota can promote microbial phosphate mineralization (Tan et al., 2013). In this study, MG and GG decreased SOC content and changed the developmental environment of Proteobacteria compared with EG. Most Acidobacteriota are oligotrophic and can maintain basal activity and participate in geochemical cycles by using fewer carbon and nitrogen sources in low-nutrient stress environments (Rime et al., 2015; Sun et al., 2021; Venkatachalam et al., 2021). Compared with EG, the relative abundance of Acidobacteriota in GG and MG increased, indicating that they can adapt to the disturbance of grazing and mowing so that the relative abundance of Acidobacteriota that efficiently utilized nutrient resources increased, and this result is consistent with Li et al. (2023). In this study, Proteobacteria and Acidobacteriota are the dominant phylum, which is consistent with the results of previous studies (Gao et al., 2017; Zhang C. et al., 2021; Song et al., 2022; Zhu et al., 2022).

The alpha diversity of soil microbes is used to describe the microbial community of an individual or a group habitat (Lozupone and Knight, 2008). The Chao1 index and Shannon index can be used to evaluate the richness and diversity of bacterial communities, and the higher the index value, the higher the richness and diversity of bacterial communities (Xue et al., 2023). In this study, the richness index (Chao1) and diversity index (Shannon) of the soil bacterial community had no significant difference among the different utilization patterns, indicating that GG and MG did not significantly change the richness and diversity of the bacterial communities compared with EG. The Venn diagram showed that the unique OTUS in MG was the largest, indicating that the unique microbial community was the most in EG, which is consistent with the study results of Zheng et al. (2022). However, compared with EG and GG, MG had no significant effect on the diversity of the soil bacterial community, indicating that external disturbance had some but limited effect on the diversity of the soil bacterial community.

### Effects of environmental factors on the soil bacterial community under different grassland use patterns

Changes in land use will affect the soil structure and nutrient status, and the structure of soil bacterial communities will also change, while soil environmental factors are important influences on the structure of soil bacterial communities (Saleem, 2015; Melo et al., 2020). The RDA showed that soil properties explained 63.87% of the variation in the bacterial communities. This indicated that the variation in microbial community composition was highly correlated with soil properties. Previous studies have shown that soil pH is one of the key factors affecting the structure of soil microbial communities (Fierer and Jackson, 2006; Griffiths et al., 2011; Ling et al., 2016; Yun et al., 2016). In our study, the Monte Carlo test showed that pH was one of the key factors affecting the soil microbial community under different use patterns, which is consistent with the results of previous studies (Fan et al., 2019; Wang et al., 2021; Song et al., 2022). This may be due to the fact that most of the bacterial groups in this study showed a relatively narrow growth tolerance and poor growth environment in the karst area (Xue et al., 2017). In this study, pH was positively associated with Acidobacteriota, which is consistent with many studies (Dai et al., 2017; Zhang J. Y. et al., 2020; Song et al., 2022). This suggests that Acidobacteriota is closely related to pH under different grassland use patterns. Verrucomicrobiota has a sparsely linked non-ribosomal peptide synthetase system, and its gene cluster has the potential to synthesize antibiotics (Bergmann et al., 2011), which indicated that external disturbance may lead to a reduction in antibiotic synthesis in the soil, which is not conducive to plant disease resistance (Fu et al., 2022). This result confirms the finding of this study, namely that the relative abundance of Verrucomicrobiota in GG and MG was decreased compared to EG. Soil AP and TP were closely related to Verrucomicrobiota, indicating that soil phosphorus played an important role in the establishment of Verrucomicrobiota. Gemmatimonadota can convert various sugar molecules into vitamins (Xu et al., 2020), and the relative abundance of Gemmatimonadota in EG was decreased in this study.

The functional diversity of microorganisms is fundamental to ensuring that all basic soil functions perform as expected (Eduardo et al., 2015; Ma et al., 2021). The results of this study showed that the dominant bacterial group in the soil ecological function group was mainly chemoheterotrophy and aerobic chemoheterotrophy, which is consistent with Zhang et al. (2018) and Wu et al. (2021). The results of ecological function prediction in soil bacteria showed that the relative abundance of aerobic chemoheterotrophic was the highest in EG and the lowest in MG. This indicated that a large number of litter were not easily used by plants in EG, and a large number of chemoheterotrophic bacteria were produced, but the litter could decompose in time in MG, which decreased the relative abundance of aerobic chemoheterotrophic.

## Conclusion

It is estimated that more than one-third of the world's soils are in degraded conditions (Wall and Six, 2015). This study was conducted on the effects of different grassland utilization patterns on soil bacterial communities in a special land-degraded landscape (karst desertification) area in the subtropical monsoon zone of China. We investigated the effects of grazing, mowing, and enclosure on the soil properties and the structure and diversity of soil bacterial communities in the artificial grassland. We drew the following conclusions: (1) Compared with EG, GG significantly changed soil pH, increased alkaline hydrolyzable nitrogen content, and decreased soil total phosphorus content. However, MG significantly decreased the contents of soil organic carbon, total phosphorus, available nitrogen, ammonium nitrogen, β-1,4-glucosidase, and N-acetyl-β-D-glucamosonidase (*P* < 0.05). (2) Different grassland use patterns significantly changed the structure of the soil bacterial community but did not significantly change the richness and diversity of the soil bacterial community. (3) Proteobacteria (29.88%) and Acidobacteriota (29.46%) were the dominant phyla. EG significantly decreased the relative abundance of Acidobacteriota and Gemmatimonadota and significantly increased the relative abundance of Proteobacteria and chemoheterotrophy. (4) pH was the main factor affecting the bacterial community structure, which is consistent with the results of the study conducted by Bahram et al. (2018) on a global scale. In this study, different grassland utilization patterns significantly altered the structure of soil bacterial communities but did not significantly alter the richness and diversity of soil bacterial communities, which may be related to the short observation period. Future studies should continue long-term observations to identify suitable grassland use patterns in the karst desertification control areas and to provide theoretical support for the ecological restoration of the karst desertification environment and the sustainable development of grassland ecosystems.

## Data availability statement

The datasets presented in this study can be found in online repositories. The names of the repository/repositories and accession number(s) can be found below: https://www.ncbi.nlm.nih.gov/, PRJNA957490.

## Author contributions

YC, KX, and SS performed the sampling, contributed to the experimental design, analyzed the data, and wrote the manuscript. KX provided the study site and funded the study. All authors contributed to the article and approved the submitted version.
